# Influence of Gut Microbiota-Derived Butyrate on Intestinal Uric Acid Excretion and Hyperuricemia Regulation by *Cichorium intybus* L.

**DOI:** 10.3390/ijms26136413

**Published:** 2025-07-03

**Authors:** Ying Yang, Yu Wang, Jinjian Huang, Yi Xu, Xiaoyang Yin, Zhijian Lin, Bing Zhang

**Affiliations:** School of Chinese Materia Medica, Beijing University of Chinese Medicine, Beijing 102488, China; yangying60@meheco.gt.cn (Y.Y.); 202201005@bucm.edu.cn (Y.W.); huangjj@bucm.edu.cn (J.H.); 20220941491@bucm.edu.cn (Y.X.); 20220935209@bucm.edu.cn (X.Y.); linzhijian83@126.com (Z.L.)

**Keywords:** butyrate, Intestinal Uric Acid Excretion, HUA, *Cichorium intybus* L., PPARγ-ABCG2 pathway

## Abstract

Hyperuricemia (HUA) is a metabolic disorder characterized by abnormal purine metabolism and/or reduced uric acid (UA) excretion. Chicory (*Cichorium intybus* L.), recognized in Traditional Chinese Medicine, is noted for its anti-HUA effects, particularly in enhancing intestinal UA excretion, though the underlying mechanisms remain unclear. Studies indicate that disruptions in gut microbiota and its metabolites are associated with HUA, and chicory has been demonstrated to ameliorate gut microbiota dysbiosis. Among gut microbiota-derived metabolites, butyrate, a short-chain fatty acid, plays a crucial role in gut functions and is linked to HUA. Therefore, butyrate may be pivotal in elucidating the mechanism by which chicory promotes intestinal UA excretion. This study aims to investigate whether chicory facilitates intestinal UA excretion through gut microbiota-derived butyrate and to elucidate the underlying mechanism. We employed an integrated methodology combining network biology with the NHANES database analysis to explore the pathological relationship between butyrate and HUA. Our findings were subsequently validated through animal experiments. We administered chicory to rats with HUA to ascertain whether butyrate serves as the key gut microbiota metabolite through which chicory promotes intestinal UA excretion. Furthermore, we utilized western blotting to assess the expression of core targets within the PPARγ-ABCG2 pathway associated with butyrate under conditions where animals received butyrate supplements and PPARγ agonists separately. The network biology indicates that butyrate is a crucial short-chain fatty acid influencing HUA. Analyses of NHANES data and animal experiments further confirm a significant negative correlation between butyrate and serum uric acid (SUA) levels. HUA rats exhibited intestinal barrier damage, impaired intestinal UA excretion, reduced butyrate levels, and decreased expression of PPARγ and ABCG2 proteins. Intervention with chicory in HUA rats repaired intestinal barrier damage, enhanced intestinal UA excretion, and increased both butyrate levels and the expression of PPARγ and ABCG2 proteins. Similarly, interventions with butyrate supplements or PPARγ agonists in HUA rats effectively promoted intestinal UA excretion and increased the expression of PPARγ and ABCG2 proteins. This study demonstrates that butyrate is a key metabolite produced by gut microbiota, through which chicory regulates gut microbiota to enhance intestinal UA excretion. The underlying mechanism involves the activation of the PPARγ-ABCG2 pathway, which is facilitated by elevated butyrate levels in the intestine.

## 1. Introduction

Hyperuricemia (HUA) is a chronic metabolic disorder resulting from abnormal purine metabolism, which is primarily characterized by elevated serum uric acid (SUA) levels. Prolonged elevation of uric acid (UA) in the body can lead to various complications, including gouty arthritis and urate nephropathy, significantly impacting human health [1,2]. The extant research has identified insufficient UA excretion as a primary factor contributing to HUA [3,4]. Currently, UA-lowering medications primarily focus on inhibiting UA production in the liver or enhancing its excretion via the kidneys. However, the intestines, which play a significant role in UA excretion, account for approximately one-third of the body’s UA elimination, serving as a crucial complement to renal UA excretion. In recent years, this important role of the intestines has attracted considerable attention from researchers.

Previous studies have uncovered a range of pathological phenomena in the intestines associated with HUA, including gut microbiota dysbiosis, intestinal barrier damage, and abnormal expression of intestinal UA transport proteins [5,6]. Compared to the intestinal barrier and UA transport proteins, the gut microbiota is often referred to as the “second largest digestive organ” in the body, playing a crucial role in maintaining intestinal homeostasis and overall metabolism [7]. Given the diverse species and functional complexity of gut microbiota, which predominantly participate in organismal metabolism through the production of active metabolites [8], a significant amount of research has focused on microbiota metabolites. Gut microbiota metabolites primarily consist of short-chain fatty acids (SCFAs), bile acids, and amino acids [9]. Among these, SCFAs represent a crucial category of functional metabolites that are indispensable for maintaining intestinal homeostasis within the gut microbiota [10]. They are involved in various physiological processes, including the proliferation of intestinal epithelial cells and the organismal inflammatory response, and they also play a pivotal role in intestinal UA excretion [11]. Notably, butyrate, which serves as a primary energy source for the intestines, is significant for maintaining the structural integrity of intestinal barrier tissue and facilitating intestinal UA transport functions [12,13,14]. It has been reported to be implicated in the progression of HUA [15].

Research has reported abnormal changes in butyrate synthesis in the intestines of gout patients with HUA, while another clinical study demonstrated that rectal administration of butyrate in healthy individuals can significantly reduce blood UA levels [16,17]. Animal experiments have also revealed a notable reduction in butyrate levels in the feces of mice exhibiting HUA [18,19]. These finding suggest that butyrate may play a pivotal role in regulating UA levels in the body. Existing literature indicates that butyrate functions as a signaling molecule involved in metabolic regulation. Its primary mechanisms of action include recognition by G protein-coupled receptors (GPCRs), peroxisome proliferator-activated receptors (PPARs), and the inhibition of histone deacetylases (HDACs) [20]. Among these mechanisms, peroxisome proliferator-activated receptor gamma (PPARγ), a vital nuclear receptor, has emerged as a potential transcriptional regulator of the ATP-binding cassette subfamily G member 2 (ABCG2), a high-capacity UA transporter protein [21]. This suggests that butyrate may act as a ligand for PPARγ, directly activating it to enhance the expression of ABCG2. Consequently, this could facilitate the regulation of ABCG2 expression and promote the excretion of UA in the intestine. However, these hypotheses necessitate further validation and in-depth investigation to confirm their validity.

*Cichorium intybus* L., commonly known as chicory, is a perennial herb belonging to the Asteraceae family. Chicory holds a significant place in Traditional Chinese Medicine and is also utilized by the Uighur people, where it is renowned for its effectiveness in invigorating the spleen and eliminating dampness. Our previous research has confirmed chicory’s UA-lowering effect and further delved into its mechanism of action, exploring aspects such as gut microbiota regulation, intestinal damage repair, and the modulation of UA transport proteins [22,23]. Chemical composition analysis of chicory conducted in prior studies revealed that chicory root is abundant in polysaccharides [24,25]. These polysaccharides offer substantial benefits in regulating gut microbiota and metabolites, providing an explanation for the positive effects observed in our earlier work regarding the improvement of intestinal microbiota structure in HUA animals treated with chicory extract [26,27]. Considering the role of gut microbiota metabolites, particularly butyrate, in the progression of HUA, the mechanism by which chicory lowers UA levels through gut microbiota metabolites warrants further elucidation.

**Figure 1:**
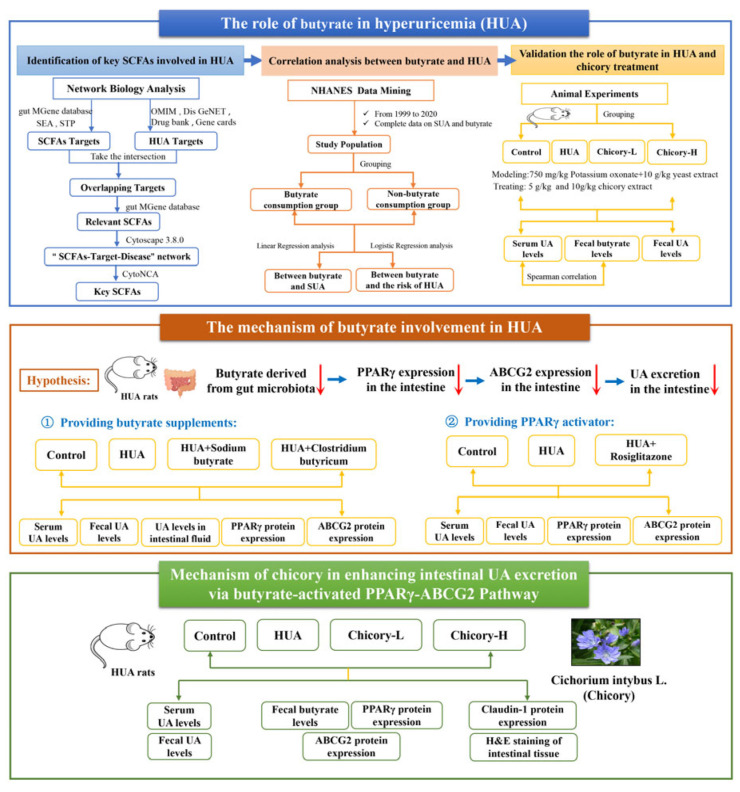
Flowchart of the technical route.

In the present study, we investigate the mechanism underlying the UA-lowering effect of chicory, with a particular emphasis on the role of butyrate. To achieve this, we adopt a comprehensive approach that integrates multiple methodologies. Initially, we first combine network biology, analysis of the NHANES, and biological experiments to examine the correlation between butyrate and HUA. Following this, we conduct animal experiments to evaluate whether butyrate can act as a key metabolite of gut microbiota contributing to chicory’s therapeutic effect on HUA to evaluate its pharmacological role in HUA treatment. Subsequently, we perform biological experiments using tool drugs, namely, butyrate supplements and PPARγ agonists, to assess the expression of core targets within the PPARγ-ABCG2 pathway associated with butyrate. The flowchart illustrating our technical approach is presented in Figure 1.

## 2. Results

### 2.1. Identification of Key SCFAs Associated with HUA Based on Network Biology Approach

#### 2.1.1. Overlapping Targets Between SCFAs and HUA

**Figure 2:**
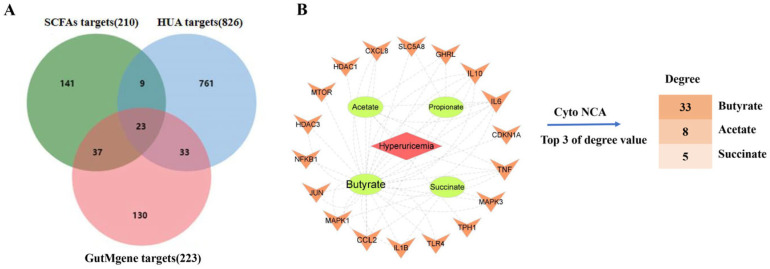
Screening of key SCFAs in HUA based on network biology. (**A**) Overlapping targets between SCFAs and HUA. (**B**) The STD Network of HUA and its topological analysis. Notes: In Figure 2A, green circles represent SCFAs targets, blue circles represent HUA targets, and red circles represent GutMgene targets. In Figure 2B, red diamonds (nodes) represent diseases, green circles (nodes) represent SCFAs, orange arrows (nodes) represent targets, and edges indicate relationships between nodes.

This study gathered seven SCFAs, including acetate, butyrate, propionate, succinate, formate, isovalerate, and isobutyrate, along with their associated 210 targets. Concurrently, 826 disease targets related to HUA were collected. An intersection analysis revealed 32 overlapping targets between the SCFAs and HUA. To refine this list further, these 32 overlapping targets were cross-referenced with 223 known gut microbiota metabolite targets from the gutMGene database. Ultimately, we identified 23 critical common targets between the SCFAs and HUA (Figure 2A).

#### 2.1.2. The SCFAs–Targets–Disease (STD) Network Analysis

The STD network illustrates the mutual relationships among SCFAs, their targets, and HUA. Notably, the targets TLR2, SLC22A6, PTGS2, G6PD, and FABP2 lacked corresponding SCFAs in the gutMGene database. Consequently, this network comprises 23 nodes, including four SCFAs associated with HUA and 18 HUA-related targets, which are interconnected by 50 edges (Figure 2B). Based on the degree centrality scores within this network, butyrate stands out as a pivotal SCFA, achieving a score of 33, which indicates the highest degree of centrality. This suggests that butyrate may play a crucial role in the pathogenesis of HUA.

### 2.2. Correlation Analysis Between Butyrate and HUA Based on the NHANES Database

#### 2.2.1. Baseline Characteristics of Participants

This section included 54,999 study subjects categorized based on butyrate consumption. Among them, 53,915 participants were in the butyrate consumption group, while 1084 were in the non-butyrate consumption group. Detailed information about the other study populations is provided (Table 1). Overall, the SUA levels in the butyrate consumption group were lower than those in the non-butyrate consumption group. No significant differences were observed in terms of age, BMI, laboratory data, diabetes, hypertension, or other comorbidities between the two groups. In comparison to the non-butyrate consumption group, the butyrate consumption group had a higher proportion of female participants, with the largest proportion being non-Hispanic white, and a lower percentage of individuals with hyperlipidemia.

#### 2.2.2. Correlation Analysis Between Butyrate Consumption and SUA Levels

The linear regression analysis examined the relationship between butyrate consumption and SUA levels. Regardless of the consideration of covariates, a statistically significant negative correlation was observed between butyrate consumption and SUA levels in both the entire population and the HUA population (Table 2). Furthermore, this negative association persisted even when potential confounding variables, such as age and gender, were disregarded; the quantity of butyrate consumption remained significantly inversely related to the SUA levels (Table 3). Notably, in Model 3, a precise quantification emerged: for each additional 1 g increase in butyrate consumption, there was a corresponding decrease of 2.11 μmol/L in SUA levels. This finding underscores the potential beneficial effect of increased butyrate consumption on reducing SUA levels.

#### 2.2.3. Correlation Analysis Between Butyrate Consumption and the Risk of HUA

The relationship between butyrate consumption and the incidence of HUA was evaluated. Logistic regression analysis indicated that, compared to the non-butyrate consumption group, the butyrate consumption group exhibited a significantly reduced risk of developing HUA (*p* < 0.001). Furthermore, even when considering covariates such as age and gender, butyrate consumption was identified as a protective factor against HUA. An odds ratio (OR) greater than 1 signifies a risk factor, while an OR less than 1 indicates a protective factor. In Model 1, for each 1.01 g increase in butyrate consumption, the odds of developing HUA decreased to 0.65 relative to not developing HUA (Table 4). Additionally, we categorized the butyrate consumption into quartiles to further examine the risk of HUA. We observed that higher consumption of butyrate was associated with a lower risk of HUA compared to the reference group (Q1) (Table 5). It appears that butyrate consumption serves as a protective factor against HUA, irrespective of factors such as age and gender.

### 2.3. Validation the Role of Butyrate in HUA Based on Animal Experiments and Its Modulation by Chicory Treatment

#### 2.3.1. Chemical Component Analysis of Chicory Extract

**Figure 3:**
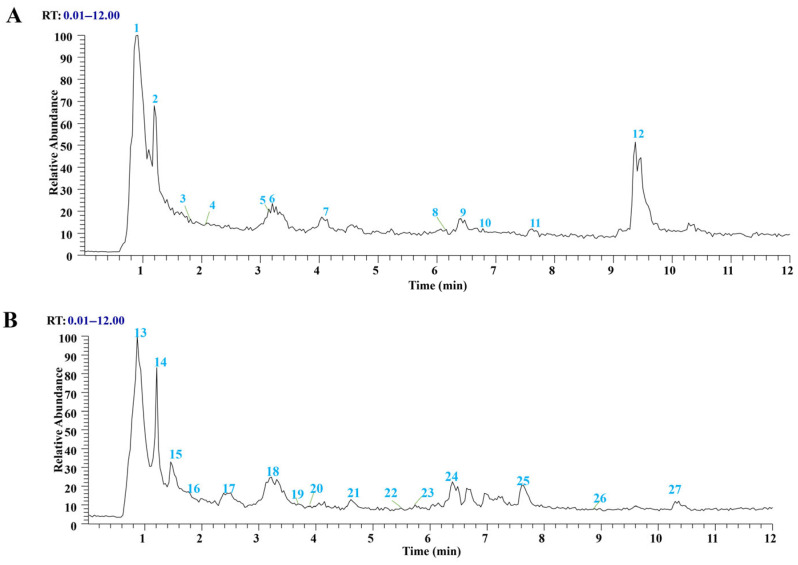
UPLC-MS ion chromatogram profile of chicory extract: (**A**) the negative ion mode; (**B**) the positive ion mode.

UPLC-MS analysis was performed to identify the chemical components of chicory extract (Figure 3). Based on the comparison of retention time, MS data, and previous literature reports, a total of 27 components were identified, including chlorogenic acid, caffeic acid, and so on (Table 6).

#### 2.3.2. Fecal Butyrate Levels and SUA Levels in HUA Rats and Chicory-Treated Rats

**Figure 4:**
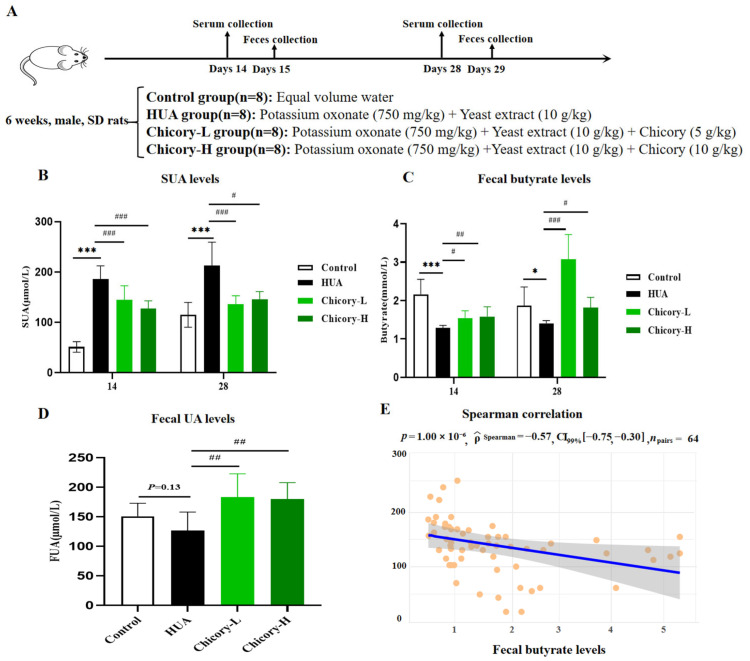
The correlation analysis between fecal butyrate levels and SUA levels in HUA rats and its modulation by chicory treatment. (**A**) The experimental protocol. (**B**) Dynamic observation of SUA levels (n = 8). (**C**) Dynamic observation of fecal butyrate levels (n = 8). (**D**) Fecal UA levels on day 28 (n = 8). (**E**) Correlation Analysis between SUA levels and butyrate levels. Compared with the control group, * *p* < 0.05 and *** *p* < 0.001. Compared with the HUA group, # *p* < 0.05, ## *p* < 0.01, and ### *p* < 0.001. Notes: In Figure 4E, each yellow circle represents a uric acid data point.

Throughout the experimental duration, the SUA and fecal butyrate levels were dynamically assessed every 14 days (Figure 4A). From day 14 until the end of the experiment, the SUA levels in the HUA group were significantly elevated compared to those in the control group (Figure 4B). Concurrently, the fecal butyrate levels in the HUA group exhibited a notable decline relative to the control group (Figure 4C). A subsequent Spearman correlation analysis revealed a pronounced negative association between SUA and fecal butyrate levels (Figure 4E). This finding suggests a potential role for butyrate in modulating UA metabolism, corroborating results from network biology and NHANES analysis. Interventions aimed at modulating butyrate levels may offer promising therapeutic strategies for HUA.

Our previous studies have reported that chicory has the ability to regulate the gut microbiota and alleviate SUA levels. Combined with the obvious role of butyrate in HUA, we observed significantly increased fecal butyrate levels and decreased SUA levels in the chicory-treated groups, along with increased fecal uric acid (FUA) levels in the chicory-treated groups (Figure 4B–D), which suggests that more butyrate production and more promotion of UA excretion in the intestine may contribute to the UA-lowering effect of chicory.

#### 2.3.3. The Mechanism of Butyrate Involvement in HUA Rats

**Figure 5:**
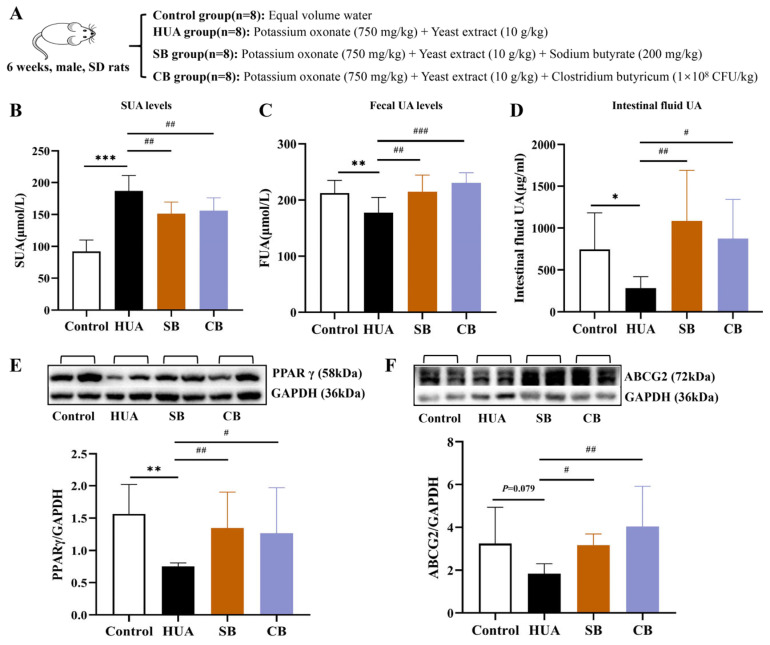
Effect of butyrate supplements in HUA rats. (**A**) Experimental protocol. (**B**) SUA levels (n = 8). (**C**) Fecal UA levels (n = 8). (**D**) UA levels in intestinal fluid (n = 8). (**E**) PPAR γ protein expression levels in intestine tissue (n = 6). (**F**) ABCG2 protein expression levels in intestine tissue (n = 6). Compared with the control group, * *p* < 0.05, ** *p* < 0.01, and *** *p* < 0.001. Compared with the HUA group, # *p* < 0.05, ## *p* < 0.01, and ### *p* < 0.001.

To confirm the role of butyrate in HUA, we administered various types of butyrate supplements, including clostridium butyricum and sodium butyrate, to HUA rats via gavage (Figure 5A). The results showed that, compared to the HUA group, the SUA levels in both butyrate supplement groups were significantly reduced throughout the experimental period, while the fecal UA levels and UA concentrations in intestinal perfusion fluid samples were significantly increased (Figure 5B–D). To explore the mechanism by which butyrate influences HUA, we assessed the protein expressions of PPARγ, a key target of butyrate, and ABCG2, a crucial uric acid transporter. The results exhibited a significant increase in the expression levels of PPARγ and ABCG2 in the intestines of both butyrate supplement groups compared to the HUA rats (Figure 5E,F).

**Figure 6:**
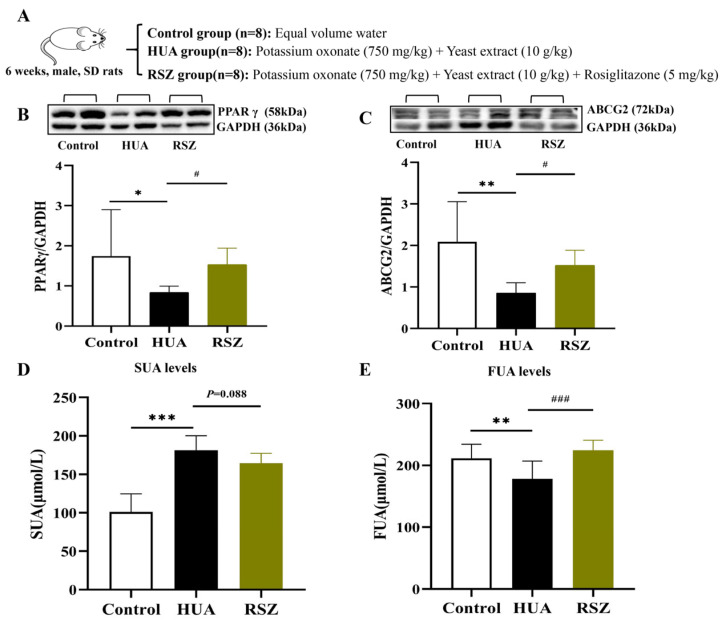
Effect of PPARγ agonist in HUA rats. (**A**) Experimental protocol. (**B**) PPAR γ protein expression levels in intestine tissue (n = 6). (**C**) ABCG2 protein expression levels in intestine tissue (n = 6). (**D**) Fecal UA levels (n = 8). (**E**) SUA levels (n = 8). Compared with the control group, * *p* < 0.05, ** *p* < 0.01, and *** *p* < 0.001. Compared with the HUA group, # *p* < 0.05 and ### *p* < 0.001.

According to research that activating PPARγ expression may enhance ABCG2 expression [21], we administered rosiglitazone, a PPARγ agonist, to HUA rats to observe alterations in ABCG2 expression, as well as SUA and fecal UA levels, in this study (Figure 6A). The results revealed that rosiglitazone significantly increased ABCG2 expression in the intestines of HUA rats, elevated the fecal UA levels, and reduced the SUA levels (Figure 6B–E). These finding confirm that the activation of the PPARγ-ABCG2 pathway significantly promotes intestinal UA excretion.

Collectively, the increase in butyrate levels within the intestine plays a critical role in the treatment of HUA, primarily through its mechanism of enhancing UA excretion via the activation of the PPARγ-ABCG2 pathway.

#### 2.3.4. Activation of the PPARγ-ABCG2 Pathway Through Increased Butyrate in Chicory-Treated Rats

**Figure 7:**
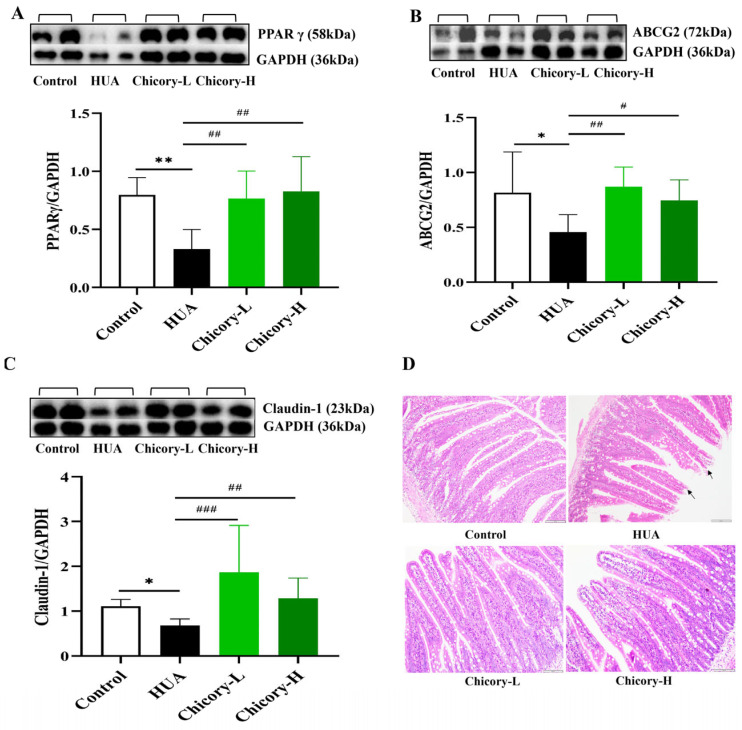
The expression levels of PPARγ, ABCG2, and claudin-1, as well as the histological analysis of intestinal tissue. (**A**) PPAR γ protein expression levels in intestinal tissue (n = 6). (**B**) ABCG2 protein levels in intestinal tissue (n = 6). (**C**) Claudin-1 protein levels in intestinal tissue (n = 6). (**D**) H&E staining of intestinal tissue captured at ×200 magnification. Compared with the control group, * *p* < 0.05, ** *p* < 0.01. Compared with the HUA group, # *p* < 0.05, ## *p* < 0.01, and ### *p* < 0.001. Notes: The scale bar in Figure 7D indicates a length of 100 μm. The area indicated by the arrow exhibits pathological alterations in the intestinal tissue.

We focused on the PPARγ-ABCG2 pathway to investigate the mechanism by which chicory enhances the intestinal excretion of UA through the modulation of butyrate. Our results revealed that, compared to the control group, the expression of PPARγ and ABCG2 in the intestines of HUA rats was significantly reduced, and following the intervention with chicory, there was a significant increase in the expression of PPARγ and ABCG2 in the intestines of HUA rats (Figure 7A,B). Combining the activating effect of butyrate on PPARγ-ABCG2 pathway and the significantly increased fecal butyrate level in chicory groups (Figure 4C and Figure 5E,F), this suggests that chicory may enhance the expression of PPARγ and ABCG2 by elevating intestinal butyrate content, thereby facilitating UA excretion in the intestine.

Additionally, we examined claudin-1, a type of intestinal barrier protein, along with a histological analysis of H&E-stained tissue, to evaluate the impact of chicory on the pathological alterations in intestinal tissue. The results revealed that the expression level of claudin-1 protein in the intestinal tissues of rats in the HUA group was significantly decreased and was accompanied by pathological phenomena such as disrupted intestinal villi structure and cell shedding. In contrast, the levels of intestinal claudin-1 protein expression were significantly increased in the chicory groups, alleviating intestinal tissue damage (Figure 6C,D).

## 3. Discussion

### 3.1. The Role of Butyrate in HUA

Hyperuricemia (HUA) is a globally prevalent condition, particularly in developed and rapidly developing nations, significantly impairing the quality of life for affected individuals. According to the NHANES conducted in the United States, the prevalence of HUA was reported as 20.2% in males and 20.0% in females during the period of 2015–2016 [28]. In Australia, the prevalence of HUA ranges from 10.5% to 16.6% [29], while in Finland, it reaches as high as 48% among the elderly population [30]. In China, HUA has emerged as the fourth most common metabolic disorder, following hyperglycemia, hypertension, and hyperlipidemia [31], affecting approximately 170 million individuals [32]. Numerous studies have indicated that the gut microbiota plays a crucial role in the pathogenesis of HUA [6,33], with dynamic changes in the microbiota observed as the disease progresses. A clinical study demonstrated there are significant differences in the gut microbiota between asymptomatic HUA patients, gout patients, and healthy individuals, as well as notable variations between the HUA and gout stages [15]. Another clinical investigation revealed that fecal microbiota transplantation from healthy donors to gout patients can significantly lower serum uric acid levels and reduce both the frequency and duration of gout attacks [34]. However, the specific regulatory mechanisms involved in these processes remain poorly understood. Recently, researchers have turned their attention to short-chain fatty acids, which are metabolites produced by gut microbiota that play a crucial role as mediators and foundational substances for the interaction between the gut microbiota and the host [35].

Butyrate is a crucial short-chain fatty acid produced by gut microbiota, serving as the primary energy source for the intestine and playing a key role in maintaining intestinal stability [36]. It has been reported to be involved in the progression of HUA [15]. Animal experiments have demonstrated a significant decrease in butyrate levels in the feces of animals with HUA [37], and supplementation with butyrate-producing bacteria can significantly reduce SUA levels [38]. These findings suggest that butyrate, an important metabolite of gut microbiota, may play a role in regulating UA levels in the body. However, further comprehensive and systematic research is necessary to elucidate the role of butyrate in HUA.

Therefore, we integrated network biology, NHANES dataset analysis, and animal experiments to systematically examine the role of butyrate in HUA. Specifically, we employed network biology to integrate the influence of short-chain fatty acids on key pathological targets associated with HUA. Through network construction and topological analysis, we identified butyrate as a pivotal metabolite implicated in the pathogenesis of HUA. Concurrently, correlation analyses of representative cohorts from the NHANES database indicated the potential therapeutic benefits of increased butyrate consumption in reducing SUA levels. This finding is supported by clinical studies demonstrating that gout patients, who are characterized by elevated SUA levels, exhibit impaired butyrate synthesis in the gut [17]. Moreover, rectal administration of butyrate has been shown to significantly reduce SUA levels [16]. However, further details regarding the clinical application of butyrate for SUA reduction require additional human intervention trials or more confirmatory experiments.

To further validate these observations, we dynamically monitored elevated SUA levels and decreased fecal butyrate concentrations in HUA rats. Additionally, we administered butyrate supplements, including clostridium butyricum and sodium butyrate, to intervene in HUA rats, and observed a significant reduction in SUA levels throughout the experimental period. Clostridium butyricum is an anaerobic, butyrate-producing bacterium that ferments undigested dietary fiber in the gut to produce butyrate [39]. Clinical studies have reported that the combination of clostridium butyricum and febuxostat produced a significantly greater UA-lowering effect compared to febuxostat alone after 28 days of treatment in patients with non-acute gout, suggesting a role of clostridium butyricum in UA reduction [40]. Sodium butyrate, the sodium salt of butyric acid, dissociates in the body to directly provide butyrate, serving as an exogenous butyrate supplement [41]. According to studies, sodium butyrate could significantly reduce SUA levels in HUA mice following a 21-day intervention [13]. These findings suggest that butyrate may act as a protective agent against the development of HUA, corroborating the results from the network biology and NHANES dataset analysis.

### 3.2. The Mechanism of Butyrate Involvement in HUA

Abnormal UA production and impaired UA excretion are the two primary causes contributing to HUA. The research indicates that approximately 90% of HUA patients exhibit impaired UA excretion, with the kidneys and intestines playing crucial roles in this process [4]. While extensive research has focused on renal excretion pathways, the intestines are emerging as a novel target organ for the development of drugs aimed at lowering SUA levels, garnering increasing attention from researchers. For instance, studies have shown that medicinal carbon tablets may lower SUA levels by facilitating intestinal excretion of UA through adsorption [42]. This finding highlights the potential of the intestinal UA excretion pathway in the prevention and treatment of HUA.

Studies have reported disrupted gut microbiota homeostasis and compromised intestinal barrier integrity in HUA [26,43,44]. Butyrate, a metabolite derived from gut microbiota, serves as the principal energy source for intestinal epithelial cells [45,46]. It plays a crucial role in maintaining intestinal barrier stability. In HUA rats, we observed a significant decrease in fecal butyrate levels, alongside a notable reduction in UA levels in the intestinal fluid and a significant decrease in claudin-1 protein expression, a respective tight junction protein in the intestine [47]. Coupled with histopathological changes in the intestinal tissue, these findings suggest that the reduction in butyrate in the intestine may leads to abnormal intestinal UA excretion, thereby promoting the development of HUA.

Therefore, the specific mechanism underlying insufficient butyrate production in HUA and its impact on intestinal UA excretion requires more in-depth investigation. Several studies have examined the effects of various short-chain fatty acids on intestinal ABCG2 expression, identifying butyrate as the most potent activator of ABCG2 [48]. Concurrently, research has shown that feeding HUA animals sodium butyrate significantly upregulated intestinal ABCG2 mRNA expression [14]. ABCG2 serves as a high-capacity UA transporter predominantly located on the apical membrane of the intestinal epithelial cells [47,49]. Multiple studies have confirmed the critical role of ABCG2 in regulating intestinal UA excretion, with its dysfunction recognized as a primary cause of HUA [50,51]. Many investigations have sought to elucidate the underlying mechanisms, revealing that PPARγ may act as a potential transcriptional regulator of ABCG2 [21,52]. Recent findings indicate that PPARγ plays a significant role in HUA and suggest its potential as a biomarker for predicting HUA [53]. Subsequent studies have reported the regulatory mechanisms of resveratrol and bergenin in modulating renal UA excretion via the PPARγ-ABCG2 signaling pathway [54,55]. Notably, PPARγ is a ligand-dependent nuclear receptor factor that is highly expressed in intestinal epithelial cells and can be directly activated by butyrate as its ligand [56]. All of the above findings indicate that PPARγ may be a key target through which butyrate regulates ABCG2 to participate in the regulation of UA excretion in the intestine. To validate this hypothesis, we observed a significant decrease in the expression of PPARγ and ABCG2 proteins in the intestinal tissues of HUA rats. Subsequently, by administering butyrate supplements to HUA rats to elevate their butyrate levels, we found a significant increase in the expression of PPARγ and ABCG2 proteins in the intestines. Furthermore, the administration of rosiglitazone, an activator of PPARγ, to HUA rats resulted in a significant increase in ABCG2 protein expression in the intestines. With the significantly reduced SUA levels and the notable increase in intestinal UA excretion observed in HUA rats treated with above tool drugs, we confirm that insufficient butyrate production interferes with intestinal UA excretion through the PPARγ-ABCG2 signaling pathway, thereby contributing to HUA progression.

### 3.3. Mechanism of Chicory in Enhancing Intestinal UA Excretion via Butyrate-Activated PPARγ-ABCG2 Pathway

Chicory, recognized as a significant medicinal and edible plant, boasts a long history of cultivation and application across Europe (notably in France, Belgium, and the Netherlands), Asia (including China and India), and certain regions of Africa. During medieval Europe, chicory was commonly utilized as both a vegetable and a coffee substitute, while ancient Egyptian texts documented its medicinal and dietary uses [57,58]. In China, chicory is regarded as a traditional Chinese medicinal herb, celebrated for its efficacy in invigorating the spleen and dispelling dampness. Recent research has extensively reported the beneficial effects of chicory in regulating metabolism, as well as promoting anti-inflammation, and anti-oxidation [59,60]. Notably, with the rising prevalence of HUA, the potential role of chicory in preventing and treating HUA has attracted widespread attention. Our previous studies have demonstrated the anti-HUA effects of chicory and found that chicory extract can improve the intestinal environment in HUA-afflicted animals by correcting dysbiosis of the intestinal microbiota, repairing damage to the intestinal barrier, and enhancing the excretion of uric acid through the intestines [22,26,61]. Furthermore, previous research indicates that chicory does not influence UA metabolism in normal rats, suggesting that the UA-lowering effect of chicory may be activated exclusively in cases of HUA, while also demonstrating good safety for the liver and kidney [59].

In the present study, we observed significantly decreased SUA levels, increased intestinal UA excretion, as well as improved histopathological morphology and enhanced claudin-1 protein expression in the intestinal tissues of HUA rats treated with chicory extract. These findings confirm that the intestine serves as a crucial target organ for anti-HUA effect of chicory. Furthermore, our previous research has elucidated the influence of chicory extract on gut microbiota in HUA rats, demonstrating an increase in the abundance of beneficial bacteria such as *Bifidobacterium* and *Lactobacillus*, alongside a reduction in harmful bacteria, including *Escherichia coli* and *Coprococcus* [62]. According to the crucial role of gut microbiota-derived butyrate in HUA progression, the present study found that chicory extract significantly increased the fecal butyrate levels in HUA rats. This finding indicates that butyrate may be the key gut microbiota metabolite through which chicory extract regulates intestinal UA excretion. Based on this observation, we further noted a significant elevation in the expression levels of PPARγ and ABCG2 proteins in the intestines of HUA rats treated with chicory. This demonstrates that the PPARγ-ABCG2 pathway may be the mechanism through which chicory promotes intestinal UA excretion by increasing the levels of gut microbiota-derived butyrate. The above results provide the first evidence that chicory promotes intestinal urate excretion through the butyrate-PPARγ-ABCG2 pathway. However, further research is warranted. For instance, in relation to the mechanism, studies employing pharmacological or genetic antagonists of PPARγ may strengthen the causal evidence, although our experiments have utilized PPARγ agonists and successfully confirmed this pathway. For the clinical translation of this study, the therapeutic efficacy of chicory in treating HUA through enhanced butyrate production needs to be validated in future clinical studies. This validation should encompass dietary equivalence, the feasibility of achieving optimal butyrate or chicory intake levels, and longitudinal monitoring of gut microbiota dynamics, as well as interindividual variations in butyrate production during chicory intervention in HUA patients.

## 4. Materials and Methods

### 4.1. Network Biology Analysis of SCFAs Involved in HUA

#### 4.1.1. Collection of HUA and SCFAs Targets

SCFAs and their associated targets were obtained from the gutMGene v1.0 section of the gutMGene database (http://bio-annotation.cn/gutmgene) accessed on 29 December 2022. All metabolites and targets of gut microbiota documented in the gutMGene database have been previously reported [63]. The Simplified Molecular Input Line Entry System (SMILES) format for SCFAs was acquired from the PubChem website (https://pubchem.ncbi.nlm.nih.gov/) accessed on 10 January 2023. This information was subsequently input into both the Similarity Ensemble Approach (SEA, https://sea.bkslab.org/) databaseand the Swiss Target Prediction database (Swiss, http://swisstargetprediction.ch/), each accessed on 15 January 2023, to predict the targets of SCFAs.

The targets related to HUA were selected from publicly accessible online databases, including the Online Mendelian Inheritance in Man (OMIM, http://omim.org), the Gene-Disease Associations database (DisGeNET, https://www.disgenet.org/), the Drugbank database (https://go.drugbank.com/), and the GeneCards database (https://www.genecards.org/), each accessed on 30 December 2022. The search term used was “Hyperuricemia”.

#### 4.1.2. Overlapping Targets of SCFAs and HUA

To identify the overlapping targets between SCFAs and HUA, we intersected the targets associated with each. Subsequently, we further intersected these overlapping targets with the 223 known human intestinal microbiota metabolite targets recorded in the gutMGene database. This process ultimately yielded the overlapping targets of SCFAs that influence HUA.

#### 4.1.3. Construction of the “SCFAs–Targets–Disease” (STD) Network

Employing the gutMGene database, we identified the SCFAs related to the key targets. Using Cytoscape 3.8.0 software, we visualized the associations among SCFAs, targets, and diseases. Additionally, we utilized CytoNCA to calculate the Betweenness centrality scores for the SCFAs, selecting the metabolite with the highest score as the key SCFA.

### 4.2. Correlation Analysis Between Butyrate and HUA Based on the NHANES Database

#### 4.2.1. Data Source

The National Health and Nutrition Examination Survey (NHANES) conducted by the National Center for Health Statistics (NCHS) under the Centers for Disease Control and Prevention (CDC) represents a comprehensive and unique resource for understanding the health and nutritional status of the U.S. population [64]. NHANES conducts periodic cross-sectional surveys on nationally representative samples, collecting extensive data on chronic diseases. All NHANES research protocols have been approved by the NCHS Institutional Review Board, and written informed consent has been obtained from all participants. Detailed information about NHANES is available on its official website.

#### 4.2.2. Study Population

This aim of this study was to investigate the correlation between butyrate levels and SUA levels, as well as the associated risk of HUA. To achieve this, we selected participants from the NHANES database spanning the years 1999 to 2020 who had complete data on dietary butyrate and SUA, comprising our study population.

#### 4.2.3. Data Extraction and Definition

Extraction of dietary butyrate intake: The dietary survey section of the NHANES database collects information on the types and quantities of beverages and foods consumed by participants within the past 24 h. From these data, the intake of various nutrients in the diet is calculated. For the purposes of this study, the butyrate content in the diet was extracted for further analysis.

Extraction of SUA and definition of HUA: SUA levels in the NHANES database are measured using the uricase peroxidase method. This study extracted SUA levels for further research. According to the consensus researched by multi-disciplinary experts on the diagnosis and treatment of HUA-related diseases in China (2023 edition), SUA levels exceeding 420 μmol/L, regardless of gender, are defined as HUA.

Definition of covariates: This study incorporated covariates that may potentially influence the relationship between butyrate and SUA levels. Demographic parameters included age, gender, race, and body mass index (BMI). Health risk factors taken into consideration included diabetes, hypertension, dyslipidemia, and the estimated glomerular filtration rate (eGFR).

#### 4.2.4. Analysis Methods

Descriptive statistical analysis: NHANES data analysis was conducted using R software version 4.2.1 (R Core Team, Auckland, New Zealand). Data extraction was performed by the “nhanesR” package version 0.9.1.1, and descriptive statistical analysis was conducted on the demographic and clinical data of the study population. Continuous variables were reported as the mean ± standard error (Mean ± SE), with differences between groups assessed using *t*-tests. Categorical variables were presented as percentages, along with their corresponding 95% confidence intervals (95% CI), and group differences were evaluated using chi-squared tests.

Linear regression and logistic regression analysis: Weighted logistic regression analysis was performed utilizing the “survey” package version 4.2-1. A significance level of *p* < 0.05 was employed to determine statistical significance. Study participants were stratified into two groups based on their consumption of butyrate: the butyrate intake group and the non-butyrate intake group. Initially, butyrate intake was treated as a continuous independent variable, and linear regression was utilized to investigate the relationship between butyrate intake and SUA levels. Subsequently, butyrate intake was categorized into quartiles (Q1–Q4), treating it as a categorical variable. Logistic regression analysis was then conducted, with Q1 serving as the reference group, to examine the association between varying levels of butyrate (Q2, Q3, and Q4 groups) and the risk of HUA. The primary objective of this analysis was to assess the impact of butyrate intake on SUA levels and the risk of developing HUA.

Three primary models were employed in the analysis: Model 1 (uncorrected for covariates), Model 2 (adjusted for covariates such as sex, age, and race), and Model 3 (adjusted for a comprehensive set of covariates including age, race, sex, BMI, diabetes, hypertension, dyslipidemia, and eGFR).

### 4.3. Preparation of Chicory Extract

The chicory root (Anhui Daoyuan Tang Chinese Medicine Pieces Company, Bozhou, China, Batch Number: 221001), was meticulously weighed. Initially, deionized water was added in a precise 1:10 ratio and allowed to soak for thirty minutes. Following this, the mixture underwent boiling extraction for one hour, after which it was filtered to obtain the liquid extract. This process was repeated using a 1:8 ratio of deionized water to chicory root, with another hour of extraction, and the resultant liquid extract was collected. The two batches of liquid extracts were subsequently combined and concentrated under reduced pressure, utilizing a water bath maintained at a consistent temperature of 80 °C. The extract was concentrated to achieve concentrations of 0.625 g/mL and 1.25 g/mL of raw chicory root, respectively.

### 4.4. UPLC-MS Analysis of Chicory Extract

The main chemical components in the chicory extract sample were analyzed using an ultra-high performance liquid chromatography (UHPLC) system (Vanquish™ Duo UHPLC system, Thermo Fisher Scientific, Waltham, MA, USA). The analysis utilized an ACQUITY UPLC BEH C18 column (1.7 µm, 2.1 mm× 100 mm, Waters, Milford, MA, USA) maintained at a column temperature of 40 °C. The mobile phase consisted of A—a 0.1% formic acid aqueous solution (formic acid sourced from Beijing Meirui Technology Company, Beijing, China, Batch No: 20201010)—and B—a 0.1% formic acid solution in acetonitrile (LC-MS grade acetonitrile from Merck, Darmstadt, Germany, Batch No: A998-4). The mobile phase was delivered at a flow rate of 0.4 mL/min, with a sample volume of 4 μL. The gradient elution program was structured as follows: 0–12 min (10–55% B); 12–15 min (55–85% B); 15–17 min (85–10% B); and 17–20 min (10% B).

The mass spectrum acquisition system utilized was a Thermo Scientific Q Exactive (Thermo Fisher Scientific, Waltham, MA, USA), which was equipped with an Electrospray Ionization (ESI) source operating in both positive and negative ion modes. The parameters for the electrospray source were configured as follows: The source temperature was set to 350 °C, and the ion spray voltages were 3200 V for positive mode and 3800 V for negative mode. A full scan was conducted at a resolution of 70,000 (FWHM), covering a data acquisition range of *m/z* 100–1000 Da. Additionally, secondary lysis was performed using Higher-energy Collisional Dissociation (HCD) with collision voltages of 30, 40, and 50 eV for positive mode, and 30, 50, and 70 eV for negative mode.

### 4.5. Animal Experiments

#### 4.5.1. Experimental Design

All animal experiments were approved by the Animal Ethics Committee of Beijing University of Traditional Chinese Medicine (Beijing, China; Approval No. BUCM-2023052301-2150 and BUCM-2023080409-3221). Male Sprague Dawley (SD) rats (obtained from SPF Biotechnology Company, Beijing, China), weighing 160 ± 10 g and aged 6 weeks, were raised in a standard environment, which included a 12-hour photoperiod, a room temperature of 25 ± 2 °C, and a relative humidity of 50–55%.

After a 5-day adaptive feeding period, 32 rats were randomly assigned to four groups (n = 8 each): The control group received standard feed and pure water; the HUA group was administered 750 mg/kg of potassium oxonate (Shanghai yuanye Bio-Technology Company, Shanghai, China) and 10 g/kg of yeast extract (Oxoid, Basingstoke, UK) via intragastric administration; the chicory extract groups, labeled chicory-L and chicory-H, were treated similarly to the HUA group but received corresponding doses of chicory extract (5 g/kg/d and 10 g/kg/d, respectively) via intragastric administration. Serum samples were collected on days 14 and 28 of the experiment, while fecal samples were obtained the day after serum sample collection.

Additionally, another set of 32 rats was randomly divided into four groups (n = 8 each): The control and HUA groups were treated as previously described; the clostridium butyricum (CB) group and sodium butyrate (SB) group received the same treatment as the HUA group, supplemented with 1 × 10^8^ CFU/kg of clostridium butyricum and 200 mg/kg of sodium butyrate, respectively, via intragastric administration. Serum and fecal samples were collected on days 28 and 29 of the experiment, respectively.

Furthermore, 24 rats were divided into three groups (n = 8 each): The control and HUA groups were treated as previously described; the rosiglitazone (RSG, Chengdu Hengrui Pharmaceutical Company, Chengdu, China) group received the same treatment as the HUA group, supplemented with 5 mg/kg of rosiglitazone via intragastric administration. Serum and fecal samples were collected on days 28 and 29 of the experiment, respectively.

At the end of the experiment, all rats were euthanized, and intestinal tissues were harvested for subsequent analysis.

#### 4.5.2. Detection of UA Levels in Serum, Feces, and Intestinal Fluid

The levels of serum uric acid (SUA) were quantified using an assay kit (Zhongsheng Beikong Biotechnology Company, Beijing, China) in strict accordance with the manufacturer’s instructions.

The levels of FUA were determined using methods reported in the literature with modifications [65]: Fecal samples were homogenized in phosphate-buffered saline (PBS), vortexed, and subsequently centrifuged for 10 min. A 400 µL aliquot of the supernatant was mixed with 100 µL of PBSL, which was prepared by dissolving 1 g of CLi_2_O_3_ in 280 mL of PBS. The mixture was sonicated for 2 min and then incubated at 100 °C for 30 min, followed by incubation at 37 °C for 2 h. Finally, the mixture was centrifuged for 15 min at 4 °C, and the final supernatant was collected for the detection of FUA levels using a UA assay kit (Zhongsheng Beikong Biotechnology Company, Beijing, China).

A 5 cm segment of jejunum, starting 15 cm distal to the stomach, should be taken and flushed with 0.2 mL of physiological saline. The intestinal fluid should be collected into a 1.5 mL EP tube followed by centrifuge at 4 °C. The supernatant should then be collected, filtered through membrane filter with a pore-size of 0.45 μm, and stored for subsequent analysis. The concentration of UA in the intestinal fluid samples was determined using high-performance liquid chromatography (HPLC, Shimadzu Corporation, Kyoto, Japan) with the following parameters: An Agilent Zorbax SB C18 column (4.6 mm × 250 mm, 5 μm, Agilent Technologies, Palo Alto, CA, USA); a mobile phase consisting of methanol (Thermo Fisher Scientific, Waltham, MA, USA)and 0.5% aqueous acetic acid (Tianjin Damao chemicals reagent factory, Tianjin, China) in a ratio of 10:90 (*v/v*); isocratic elution for 10 min; a flow rate of 0.6 mL/min; a column temperature set at 30 °C; a detection wavelength of 288 nm; an injection volume of 5 μL for the control and 20 μL for the sample; and quantification performed using an external standard method.

#### 4.5.3. Detection of Butyrate Content in Feces

Fresh feces samples were homogenized in a 4 mmol/L solution of 2-ethylbutyric acid (Shanghai Macklin Biochemical Company, Shanghai, China) and vortexed under ice bath conditions. After centrifugation for 10 min at 4 °C, 500 μL of the supernatant was adjusted to a pH of 2–3 using HCl (Tianjin Damao chemicals reagent factory, Tianjin, China) and vortexed again under ice bath conditions. Following a second centrifugation for 10 min at 4 °C, the supernatant was filtered through a 0.45 μm filter.

Gas chromatography analysis (Agilent Technologies, Palo Alto, CA, USA) of butyrate content in feces was conducted using a DB-FFAP column (30 m × 0.25 mm × 0.25 μm, Agilent Technologies, Palo Alto, CA, USA) with a temperature program ranging from 100 °C (10 min) to 200 °C (5 min) at a rate of 30 °C/min. The flow rate was set at 1 mL/min with an injection volume of 1 μL in split mode. The injector temperature was maintained at 250 °C, and flame ionization detection (FID) was performed at 300 °C using nitrogen (N_2_) as the carrier gas, hydrogen (H_2_) for combustion, and air as the auxiliary gas. The tail flow was set at 40 mL/min, resulting in a total run time of 18.3 min.

#### 4.5.4. Histological Evaluation

4% Paraformaldehyde (Servicebio, Wuhan, China) was added to the intestinal tissue samples and left overnight for fixation. The tissues were subsequently embedded, sectioned to a thickness of 3 μm, and stained with hematoxylin and eosin (H&E, Beijing Solarbio Science & Technology Company, Beijing, China). This staining procedure was conducted to identify histological alterations in the intestinal structure.

#### 4.5.5. Western Blotting Analysis

Western blotting was employed to assess the expression levels of claudin-1, PPARγ, and ABCG2. Intestinal tissue samples (100 mg) were homogenized in RIPA buffer supplemented (NCM Biotech, Suzhou, China) with protease and phosphatase inhibitors (NCM Biotech, Suzhou, China) to extract total protein. The protein concentrations were quantified using a BCA Protein Assay Reagent Kit (Beijing Solarbio Science & Technology Company, Beijing, China). Equal amounts of protein (60 µg) were loaded onto 10% sodium dodecyl sulfate polyacrylamide gels and subsequently transferred onto polyvinylidene fluoride (PVDF, Merck, Darmstadt, Germany) membranes via electroblotting. The membranes were blocked with 5% skimmed milk in TBST (Beijing Solarbio Science & Technology Company, Beijing, China) for 2 h and incubated overnight at 4 °C with primary antibodies against Claudin-1 (1:1000, Abclonal, Wuhan, China), PPARγ (1:1000, Abcam, Cambridge, UK), ABCG2 (1:1000, Abclonal, Wuhan, China), and GAPDH (1:50,000, Proteintech, Rosemont, IL, USA). After washing with TBST, the membranes were incubated with HRP-linked anti-rabbit IgG (1:5000, Proteintech, Rosemont, IL, USA) or HRP-linked anti-mouse IgG (1:5000, Proteintech, Rosemont, IL, USA) for 1 h at room temperature. The PVDF membranes were rinsed three times with TBST buffer for 10 min each and developed using enhanced chemiluminescence (ECL, Biorigin, Beijing, China) detection kits. Band brightness was quantified using ImageJ software, https://imagej.net (NIH, Bethesda, MD, USA).

### 4.6. Statistical Analysis

Data were analyzed using SPSS v27.0 (IBM, Armonk, NY, USA). All values are presented as means ± standard deviations. Experimental data that followed a normal distribution were analyzed with one-way ANOVA, whereas non-normally distributed data were subjected to a nonparametric test. *p* < 0.05 was considered statistically significant. Graphical visualizations were created using GraphPad Prism 8.0.1 (GraphPad Software Inc., San Diego, CA, USA).

## 5. Conclusions

In summary, this study explored the role and specific mechanism of gut microbiota-derived butyrate in the progression of HUA, employing an integrated methodology that combines network biology, the NHANES database, and animal experiments. The results indicated that a reduction in butyrate levels may lead to abnormal intestinal UA excretion through the PPARγ-ABCG2 pathway, thereby contributing to HUA progression. This finding identifies butyrate as a key metabolite of the gut microbiota in modulating intestinal UA excretion. Based on these findings, we further investigated the underlying mechanisms of chicory’s anti-HUA effects and clarified that chicory enhances intestinal UA excretion by activating the PPARγ-ABCG2 pathway through increased levels of gut microbiota-derived butyrate. The present study elucidates the mechanism by which chicory promotes intestinal UA excretion to improve HUA, providing experimental evidence for chicory as a potential therapeutic agent for HUA treatment in the future.

## Figures and Tables

**Table 1:** Characteristics of participants.

Characteristics	Overall (The Sample Size Was *n* = 54,999)	Butyrate Consumption		*p*-Value
		No	Yes	
Age Mean (SE)	43.46 (0.19)	42.41 (0.68)	43.47 (0.19)	0.12
BMI Mean (SE)	28.20 (0.06)	27.85 (0.28)	28.21 (0.06)	0.21
SUA, μmol/L Mean (SE)	318.65 (0.59)	337.79 (4.14)	318.36 (0.58)	<0.0001
Butyrate, g Mean (SE)	0.99 (0.01)	0.00 (0.00)	1.01 (0.01)	<0.0001
Gender, %				<0.001
Male	48.09 (46.32, 49.86)	54.27 (50.72, 57.83)	47.99 (47.55, 48.44)	
Female	51.91 (50.02, 53.81)	45.73 (42.17, 49.28)	52.01 (51.56, 52.45)	
Race/Ethnicity, %				<0.0001
Non-Hispanic Black	10.81 (9.88, 11.75)	22.20 (17.08, 27.33)	10.64 (9.55, 11.73)	
Non-Hispanic White	68.83 (64.67, 72.99)	46.68 (40.18, 53.18)	69.17 (67.18, 71.16)	
Mexican American	8.46 (7.50, 9.41)	10.62 (8.34, 12.90)	8.42 (7.36, 9.49)	
Other Race—Including Multi-Racial	6.31 (5.80, 6.83)	12.15 (8.75, 15.55)	6.22 (5.70, 6.75)	
Other Hispanic	5.58 (4.73, 6.44)	8.34 (3.94, 12.74)	5.54 (4.69, 6.39)	
Diabetes mellitus, %				0.36
yes	8.42 (7.97, 8.87)	7.63 (5.76, 9.50)	8.55 (8.19, 8.91)	
no	90.26 (87.00, 93.51)	92.37 (90.50, 94.24)	91.45 (91.09, 91.81)	
Hyperlipidemia, %				0.003
yes	67.96 (65.27, 70.65)	72.21 (69.65, 74.78)	67.89 (67.17, 68.62)	
no	32.04 (30.86, 33.22)	27.79 (25.22, 30.35)	32.11 (31.38, 32.83)	
Hypertension, %				0.31
yes	33.55 (32.04, 35.05)	35.66 (31.48, 39.85)	33.52 (32.71, 34.32)	
no	66.45 (64.04, 68.86)	64.34 (60.15, 68.52)	66.48 (65.68, 67.29)	
Renal function/min per 1.73 m^2^	94.04 (0.29)	93.29 (1.09)	94.05 (0.29)	0.48

Mean (SE) was for continuous variables. The percentage (95% confidence interval) was for categorical variables. SE—standard error; BMI—body mass index; SUA—serum uric acid.

**Table 2:** The association between butyrate consumption and SUA levels.

Model	HUA Population			Overall Population		
	β	95% CI	*p* Value	β	95% CI	*p* Value
Model 1	−10.59	(−4.23, −2.45)	0.029	−19.43	(−23.49, −15.37)	0.0000
Model 2	−10.13	(−2.30, −1.12)	0.037	−14.81	(−18.70, −10.91)	0.0002
Model 3	−12.22	(−3.02, −1.19)	0.010	−12.42	(−16.20, −8.63)	0.0013

**Table 3:** The association between butyrate consumption quantity and SUA levels.

Model	β	95% CI	*p* Value
Model 1	−3.34	(−4.23, −2.45)	<0.0001
Model 2	−2.06	(−2.30, −1.12)	0.03
Model 3	−2.11	(−3.02, −1.19)	0.023

**Table 4:** Correlation analysis between butyrate consumption and the risk of HUA.

Variables	Butyrate Intake		*p* Value
	No	Yes	
Butyrate consumption quantity/g	0.00 (0.00)	1.01 (0.01)	
Overall Population	1084	53,915	
HUA Population	174	6105	
Incidence Rate (95% CI), %	16.78 (13.63, 19.92)	11.59 (11.18, 12.00)	<0.001
Odds Ratios (95% CI)			
Model 1	1	0.65 (0.58, 0.73)	0.0002
Model 2	1	0.69 (0.62, 0.78)	0.0032
Model 3	1	0.68 (0.60, 0.78)	0.0037

**Table 5:** Correlation analysis between butyrate consumption quantity and the risk of HUA.

Variables	Butyrate Consumption Quantity						
	Q1	Q2	*p* Value	Q3	*p* Value	Q4	*p* Value
Butyrate consumption quantity/g	0–0.35	0.350–0.714		0.714–1.254		>1.254	
Overall Population	13,521	13,456		13,461		13,477	
HUA Population	1781	1459		1445		1420	
Incidence Rate (95% CI), %	13.13 (12.37, 13.89)	11.13 (10.38, 11.88)		11.12 (10.48, 11.75)		11.25 (10.40, 12.11)	
Odds Ratios (95% CI)							
Model 1	1	0.83 (0.79, 0.87)	<0.001	0.83 (0.79, 0.87)	<0.001	0.84 (0.80, 0.88)	0.001
Model 2	1	0.83 (0.79, 0.87)	<0.001	0.79 (0.75, 0.83)	<0.001	0.68 (0.65, 0.72)	<0.001
Model 3	1	0.82 (0.77, 0.86)	<0.001	0.76 (0.72, 0.80)	<0.001	0.65 (0.61, 0.69)	<0.001

**Table 6:** Results of UPLC-MS analysis of chicory extract.

Peak Name	Retention Time (min)	*m*/*z* (Experiment)	Reference Ion	Compound Name	Pubchem Number
1	0.928	179.0554	[M − H] − 1	β-D-Glucofuranose	34,784,518
2	1.207	173.0083	[M − H] − 1	trans-Aconitic acid	444,212
3	1.828	293.1035	[M − H] − 1	cis-diphenylglycoluril	21,237
4	2.075	353.0885	[M − H] − 1	Neochlorogenic acid	5,280,633
5	3.069	353.0883	[M − H] − 1	Chlorogenic acid	1,794,427
6	3.078	175.0604	[M − H] − 1	2-Isopropylmalic acid	5,280,523
7	4.167	257.0822	[M − H − H_2_O] − 1	lactucin	442,266
8	6.055	515.1204	[M − H] − 1	4,5-Dicaffeoylquinic acid	6,474,309
9	6.528	259.0978	[M − H] − 1	8-deoxylactucin	442,196
10	6.82	187.0971	[M − H] − 1	Azelaic acid	2266
11	8.005	200.1288	[M − H] − 1	Capryloylglycine	84,290
12	9.324	327.2183	[M − H] − 1	Corchorifatty acid F	44,559,173
13	1.161	130.0976	[M + H] + 1	4-guanidinobutanal	559
14	1.201	268.1039	[M + H] + 1	Adenosine	60,961
15	1.681	332.1338	[M + H] + 1	5′-O-β-D-Glucosylpyridoxine	440,188
16	1.986	211.0965	[M + H] + 1	3,4-Dimethoxyhydrocinnamic acid	75,019
17	2.038	163.039	[M + H − H_2_O] + 1	Caffeic acid	689,043
18	3.199	279.1227	[M + H] + 1	11β,13-dihydro-lactucin	21,578,003
19	3.729	260.1129	[M + H] + 1	Rhodiocyanoside A	6,442,274
20	3.87	199.0603	[M + H] + 1	Vanillylmandelic acid	1245
21	4.155	339.1073	[M + H] + 1	3-O-coumaroylquinicacid	9,945,785
22	5.435	425.1804	[M + H] + 1	Crepidiaside B	101,683,332
23	5.886	423.165	[M + H] + 1	Crepidiaside A	13,855,728
24	6.401	263.1278	[M + H] + 1	jacquinelin	14,163,574
25	7.631	145.1012	[M + H] + 1	4-pentynylbenzene	74,573
26	8.974	227.1278	[M + H] + 1	1,6-Hexanediol diacrylate	25,644
27	10.127	233.1535	[M + H] + 1	(+)-Alantolactone	327,378

## Data Availability

The data supporting the findings of this study are available from the corresponding author upon reasonable request.

## Abbreviations

ABCG2—ATP-binding cassette superfamily G member 2; BMI—body mass index; butyrate—butyric acid; CB—clostridium butyricum; CDC—Centers for Disease Control and Prevention; chicory—*Cichorium intybus* L.; DisGeNET—Gene-Disease Associations database; ECL—enhanced chemiluminescence; eGFR—estimated glomerular filtration rate; ESI—Electrospray Ionization; FID—flame ionization detection; FUA—fecal uric acid; GC—gas chromatography; GPCRs—G protein-coupled receptors; HCD—Higher-energy Collisional Dissociation; HDAC—histone deacetylase; H&E—hematoxylin–eosin staining; HPLC—high-performance liquid chromatography; HUA—hyperuricemia; NHANES—National Health and Nutrition Examination Survey; NCHS—National Center for Health Statistics; OMIM—Online Mendelian Inheritance in Man; OR—odds ratio; PBS—phosphate-buffered saline; PO—potassium; PPARs—peroxisome proliferator-activated receptors; PPARγ—peroxisome proliferators-activated receptors gamma; PVDF—polyvinylidene fluoride; RSG—rosiglitazone; SB—sodium butyrate; SCFAs—short-chain fatty acids; SD—Sprague Dawley; SE—standard error; SEA—Similarity Ensemble Approach; SMILES—Simplified Molecular Input Line Entry System; STD—SCFAs–Targets–Disease; SUA—serum uric acid; Swiss—Swiss Target Prediction database; UA—uric acid; UHPLC—ultra-high performance liquid chromatography.
